# Schizophrenia-Like Behaviors Arising from Dysregulated Proline Metabolism Are Associated with Altered Neuronal Morphology and Function in Mice with Hippocampal PRODH Deficiency

**DOI:** 10.14336/AD.2023.0902

**Published:** 2024-08-01

**Authors:** Yuxiao Yao, Chenchen Jin, Yilie Liao, Xiang Huang, Ziying Wei, Yahong Zhang, Dongwei Li, Huanxing Su, Weiping Han, Dajiang Qin

**Affiliations:** ^1^Key Laboratory of Biological Targeting Diagnosis, Therapy and Rehabilitation of Guangdong Higher Education Institutes, The Fifth Affiliated Hospital of Guangzhou Medical University, Guangzhou 510799, China.; ^2^Bioland Laboratory, Guangzhou Regenerative Medicine and Health Guangdong Laboratory, Guangzhou, China.; ^3^Guangzhou Laboratory, Guangzhou, Guangdong, China.; ^4^Institute of Chinese Medical Sciences, University of Macau, Macau, China.; ^5^Institute of Molecular and Cell Biology, Agency for Science, Technology and Research (A*STAR), Singapore.; ^6^Centre for Regenerative Medicine and Health, Hong Kong Institute of Science & Innovation, Chinese Academy of Sciences; Hong Kong SAR, China.

**Keywords:** hippocampus, metabolism, neuron, PRODH, proline, schizophrenia

## Abstract

Despite decades of research being conducted to understand what physiological deficits in the brain are an underlying basis of psychiatric diseases like schizophrenia, it has remained difficult to establish a direct causal relationship between neuronal dysfunction and specific behavioral phenotypes. Moreover, it remains unclear how metabolic processes, including amino acid metabolism, affect neuronal function and consequently modulate animal behaviors. PRODH, which catalyzes the first step of proline degradation, has been reported as a susceptibility gene for schizophrenia. It has consistently been shown that PRODH knockout mice exhibit schizophrenia-like behaviors. However, whether the loss of PRODH directly impacts neuronal function or whether such neuronal deficits are linked to schizophrenia-like behaviors has not yet been examined. Herein, we first ascertained that dysregulated proline metabolism in humans is associated with schizophrenia. We then found that PRODH was highly expressed in the oreins layer of the mouse dorsal hippocampus. By using AAV-mediated shRNA, we depleted PRODH expression in the mouse dorsal hippocampus and subsequently observed hyperactivity and impairments in the social behaviors, learning, and memory of these mice. Furthermore, the loss of PRODH led to altered neuronal morphology and function both *in vivo* and *in vitro*. Our study demonstrates that schizophrenia-like behaviors may arise from dysregulated proline metabolism due to the loss of PRODH and are associated with altered neuronal morphology and function in mice.

## INTRODUCTION

Schizophrenia is a severe and complex psychiatric disorder. Since 1990, its prevalence has risen from 13.1 million to more than 20 million cases in less than three decades [1, 2]. Magnetic resonance imaging studies have revealed structural abnormalities in different brain regions of schizophrenia patients, including the cerebral cortex, hippocampus, and thalamus [3-5]. In addition, autopsy results obtained from schizophrenia patients have shown a significant difference in the cellular organization of the pyramidal cell layer of the hippocampus, which is a key relay station for neural signal transmission [6]. Proton magnetic resonance spectroscopy analyses of cerebrospinal fluid and postmortem brain tissue extracts have uncovered altered glutamate concentrations in schizophrenia patients [7]. These studies, including the post-mortem findings, highlight that schizophrenia is associated with neuronal defects, such as altered synaptic protein levels and lower synaptic density [8]. Although abnormalities in the neuronal structure and function across different brain regions have been detected in patients with schizophrenia, the roles of the metabolic microenvironment and associated risk genes are poorly defined.

Amino acids are well known for their roles in supporting neuronal function in two ways; directly, by mediating neuronal communications as neurotransmitters, and indirectly, by providing energy as metabolic substrates [9]. For example, glutamate and aspartate both function as excitatory neurotransmitters, while glycine acts as an inhibitory neurotransmitter [10]. Glutamine is metabolized to produce energy through glutaminolysis, while tryptophan is converted to supply nicotinamide adenine dinucleotide [9]. However, there has been a limited number of studies regarding the neuronal regulation role played by amino acids with no known neurotransmission function, such as proline. Interestingly, depression has recently been associated with microbiota alterations that are related to proline metabolism and the accumulation of proline in the brain [11], suggesting that proline may play a role in neuronal function.

Proline participates in several critical metabolic cycles, including glycolysis, the tricarboxylic acid cycle, the urea cycle, and the pentose phosphate pathway [12]. Proline synthesis requires P5CS (pyrroline-5 carboxylate synthase) and PYCRs (pyrroline carboxylate reductases), and its degradation is catalyzed by PRODH (proline dehydrogenase) [13]. The overexpression of proline synthesis enzymes or the knockdown (KD) of its degradation enzymes leads to the accumulation of proline, which consequently leads to hyperprolinemia [14]. The first published finding that dysregulated proline metabolism is associated with CNS (central nervous system) abnormalities was a case study in 1989, which reported that a child with hyperprolinemia exhibited diffused white matter in the brain and died a few months later [15]. More recently, more than 8 single nucleotide polymorphisms (SNP) of PRODH have been linked to schizophrenia [16]. These findings implicate proline and its metabolism to be involved in neurological or psychiatric diseases, which warrants further investigation into whether the dysregulation of proline metabolism may underlie certain psychiatric disorders, such as schizophrenia.

## MATERIALS AND METHODS

### Animals

Newborn (P0) and adult C57BL/6J mice (6-8 weeks old, 22-28 g, male) were purchased from Gempharmatech Biotechnology Ltd. (Jiangsu, China). The adult mice were housed to contain 4-6 mice per cage and kept in a controlled environment (22 ± 2 °C, 45 ± 10% humidity, 12 h light/dark cycle) with free access to food and water. All experimental procedures were approved by the Laboratory Animal Ethics Committee of the Guangzhou Institutes of Biomedicine and Health.

### Stereotactic injection

After a 1-week acclimatization period, the mice were fixed in a stereotaxic apparatus (instruments obtained from RWD Life Science) and anesthetized with isoflurane (3.0% for induction and 1.5% for maintenance). AAV-*NC* and AAV-sh*Prodh* were then bilaterally injected into the hippocampus region of mice by using an automatic microinjection system (World Precision Instruments) according to the following coordinates: AP = -1.82; ML = ± 1.25; DV = -1.75. The injections were performed at a speed of 1 nL/s with a Hamilton needle (700 nL, 6.08 × 10^12^ viral particles per mL). The needle was left in place for 15 min before it was slowly retracted. Behavioral tests were conducted 3-5 weeks after the virus injection. The expression of PRODH was silenced using shRNA. Custom-made AAV vectors carrying shRNA targeting mouse *Prodh* following the U6 promoter (shRNA-*Prodh*, target site: 5’-GCA CCT ACT TCT ATG CCA A) and control AAV-*NC* (shRNA-control, target site: 5’-TTC TCC GAA CGT GTC ACG T) were purchased from Obio Technology Co., Ltd. (Shanghai, China). The mice were randomly divided into the NC (negative control) and shProdh (PRODH KD) groups. All the KD mice were confirmed by Western blot. The PRODH knockdown efficiency in the hippocampus was approximately 60-80%. Other regions, like the cerebellum, were also checked to exclude off-target effects.

### Proline measurement

Proline concentrations were measured according to the instructions supplied with the Proline Concentration Detection Kit (Solarbio, China, BC0295). Briefly, after extraction with sulfosalicylic acid (SA), proline was heated in acidic ninhydrin solution. After a second extraction with 80% methanol, the absorbance of the resulting solution was measured at 520 nm. This assay was repeated at least once more using different batches of injection mouse or transfected primary neurons.

### HPLC-mass spectrometry

The contents of proline and different neurotransmitters were measured by HPLC-mass spectrometry. Briefly, mouse brain tissues and transfected primary neurons were homogenized with ice-cold 80% methanol in water and then incubated at 4°C for 30 minutes with shaking at 1,500 rpm. The resulting lysate was centrifuged at 12,000 rpm for 15 minutes. The supernatant was then removed to another clean, 1.5 mL centrifuge tube and dried with a Speed-Vac (Fischer Scientific, USA). The extracts were redissolved with 2% acetonitrile in water and analyzed by HPLC-mass spectrometry [17]. Each group included 4 samples. No repeat batches were included for HPLC-mass spectrometry.

### Behavioral assays

Before behavioral testing, the experimental mice were kept in the behavior room for at least 1 week to adapt to the environment. Testing was conducted in a quiet, dimly lit (75 lux) room. Behaviors were tracked and analyzed by center-point detection using EthoVision XT 9 software (Noldus Information Technology, Leesburg, VA). Track visualization maps were produced with the EthoVision track generator. All the behavioral tests were repeated at least once using different batches of injected mice.

#### Open-field test (OFT)

General exploratory locomotion in an open field apparatus was evaluated within a 25 cm × 25 cm square box. During the test, a mouse was placed in the center of the open field box for 5 min of adaptation and then allowed to explore freely for another 5 min. The total distance moved (as a measure of locomotor activity) and the duration spent in the central area (as a measure of anxiety-like behavior) were analyzed with EthoVision 9 (Noldus) software.

#### Elevated plus maze (EPM) test

The elevated plus-maze test, which tests for anxiety-like behavior, was conducted as previously described [18]. The test apparatus consisted of four black polypropylene arms. The two “open” arms had 0.5 cm ledges, while the two “closed” arms had 30 cm-long walls. The open arms were placed opposite each other. The arms were 10 cm wide, 50 cm long, and were placed on 55 cm tall acrylic legs. At the beginning of each test, the mice were placed in the center of the apparatus facing an open arm. The mice were then allowed to explore the apparatus for 10 min while their behavior was recorded with a camera mounted above the maze. The EPM was divided into five zones (two open arms, two closed arms, and a center zone). The following behavioral parameters were determined: duration and number of entries into the open arms, closed arms, and center zone, and the total distance traveled.

#### Three-chamber sociability test

The three-chamber sociability test was performed as previously described [19]. The social interaction test consisted of two 10-min phases. In the first phase, a stranger mouse (stranger 1) was placed in the left chamber, while the right chamber was kept empty. A test mouse was allowed to freely explore the apparatus for 10 min. In the second phase, a new stranger mouse (stranger 2) was placed into the empty chamber. The test mouse was then allowed to explore for another 10 min. The duration and frequency of mouse entries into each chamber were analyzed by EthoVision 9 (Noldus) software.

#### Morris water maze test

The morris water maze (MWM) test was conducted essentially as previously described [20]. Briefly, the MWM was a circular pool that was 120 cm in diameter and 50 cm in height, which was filled with water. The pool was virtually divided into 4 equal quadrants. A white circular platform that was 12 cm in diameter was submerged at 1 cm under the opaque water surface in a fixed quadrant center, rendering it invisible to the mice at the water level. For 5 consecutive days, the mice performed 4 trials per day (90 s/trial) to locate the hidden escape platform. The start location was randomly assigned to one of the four quadrants of the pool. If the mice did not reach the hidden platform within the allotted time, the trial was terminated, and the mice were carefully guided to the platform. After the mice climbed onto the platform, they were left there for 30 s before being removed from the water maze. The mice were then dried and returned to their home cages until the next test. On day 7, the evacuation platform was removed, and the tests were performed.

### Immunofluorescence

**Figure 1.**
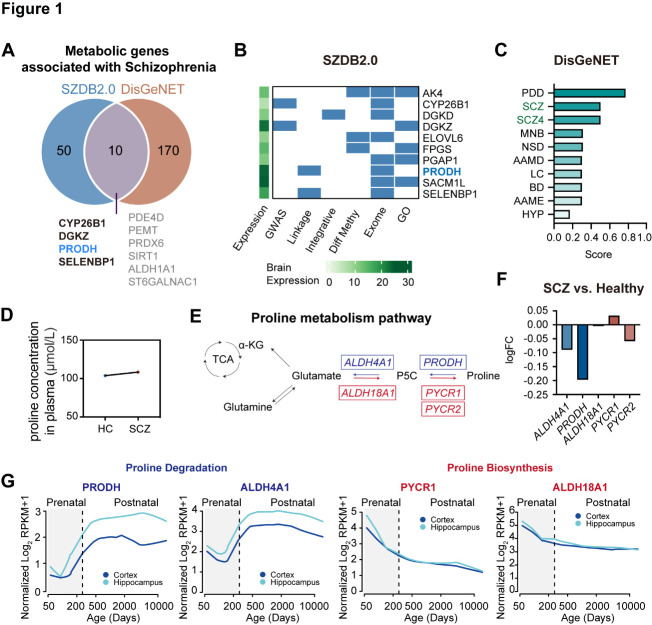
**Association between the proline metabolism pathway and schizophrenia**. (**A**) Venn diagram displaying metabolic genes associated with schizophrenia from the SZDB2.0 and DisGeNET databases. (**B**) Metabolic genes associated with schizophrenia from SZDB2.0 (differentially expressed genes with a total score ≥ 4). (**C**) Top 10 diseases associated with PRODH from DisGeNET. (**D**) Proline concentration in the human plasma of schizophrenia patients (SCZ, N = 208) and healthy controls (HC, N = 175). (**E**) Schematic diagram of the proline metabolism pathway. F: mRNA expression ratio of proline metabolism enzymes in schizophrenia patients compared to healthy controls. G: Gene expression in the human cortex and hippocampus over the entire span of neurodevelopment. PDD: Proline dehydrogenase deficiency; SCZ: schizophrenia; MNB: malignant neoplasm of the breast; NSD: nervous system disorder; AAMD: amino acid metabolism disorders, inherited; LC: liver cirrhosis, experimental; BD: bipolar disorder; AAME: amino acid metabolism errors, inborn; HYP: hyperprolinemia.

After the behavioral experiments, the mice from both groups were euthanized and pathological changes that had occurred were examined using the immunofluorescence technique (Fig. 1). The brain tissue of mice was post-fixed for 24 h in a 4% PFA solution and stored in a 15/30% gradient sucrose solution for an additional 24 h. The brains were cut coronally into 50 μm-thick sections by using a cryostat. The sections were permeabilized in PBS containing 0.01% Triton X-100 3 times, followed by incubation in 4% goat serum blocking buffer for 60 min. The sections were then incubated with rabbit anti-PRODH antibody (1:200, Proteintech, 22980-1-AP), rabbit anti-PYCR1 (1:50, Proteintech, 13108-1-AP), or rabbit anti-PYCR2 (1:50, Proteintech, 17146-1-AP) overnight at 4°C, followed by incubation with Alexa fluorescent 555-conjugated secondary antibody (1:1000, Thermofisher, A21428) and Hoechst 3342 (1:1000, Thermofisher, H1399) for 2 h at room temperature. Images were captured using a Zeiss microscope (Carl Zeiss, Jena, Germany). All immunostaining procedures included a secondary antibody-only control to distinguish genuine target staining from the background.

For cultured cells, cells were washed with PBS and then fixed with 4% PFA (with 4% sucrose). After 15 min of fixation at room temperature, the cells were incubated in a 3% BSA blocking buffer, followed by primary antibody chicken anti-MAP2 (1:2000, Abcam, ab5392) overnight at 4°C, and then Alexa fluorescent 647-conjugated secondary antibody (1:1000, Thermofisher, A32933) and Hoechst 3342 (1:1000, Thermofisher, H1399) for 2 h at room temperature. Images were captured with a Zeiss microscope (Carl Zeiss, Jena, Germany).

### Western blotting

Each mouse brain part was carefully dissected on ice using the Allen Brain Explorer software to subsequently undergo Western blotting. The dissected tissue was then weighed, cut into pieces, and homogenized in RIPA buffer (Beyotime, P0013B) supplemented with PMSF (Beyotime, ST506) and a broad-spectrum phosphatase inhibitor. Cultured cells were washed with PBS and incubated in RIPA buffer directly. The samples were then centrifuged at 13,000 rpm for 15 min at 4°C. The resulting supernatants were collected and stored at -80°C for subsequent use.

The protein concentration of the lysate was measured using a BCA analysis kit (Beyotime, P0009) before the samples were separated by 8% SDS-PAGE and transferred to PVDF membranes at a constant current of 200 mA for 90 min. The membranes were then covered with 5% bovine serum albumin (BSA) before being incubated with the primary antibody (see Table S1). After washing and incubating the membranes with secondary antibodies (anti-rabbit IgG 1:2000; anti-mouse IgG 1:2000) for 2 h at room temperature, an enzyme-linked chemiluminescence signal was developed. Finally, the resulting band intensities were quantified by using Image Lab (Bio-Rad Laboratories, United States). The relative intensities associated with mouse anti-GAPDH (1:1000, Proteintech, 60004-1-Ig), β-Actin (1:1000, Proteintech, 66009-1-Ig), and α-Tubulin (1:1000, Proteintech, 66031-1-Ig) were quantified and normalized to the control values. Each experiment was performed at least 3 times.

### Immunohistochemistry (IHC)

Frozen mouse brain sections were washed with PBS three times. The slices were then incubated in 3% H_2_O_2_ for 10 min, followed by 10% goat serum-blocking buffer. Then, the sections were incubated with rabbit anti-PRODH antibody (1:200, Proteintech, 22980-1-AP) overnight at 4°C. The sections were then incubated with a biotin-labeled secondary antibody and streptavidin-peroxidase for 30 min each, followed by development using DAB (Dako, GK500705) and light counterstaining with hematoxylin.

### Golgi-cox Staining

Mouse brains were processed for Golgi staining using the Rapid Golgi Kit (FD Neuro-Technologies, MD, PK401) according to the manufacturer's protocol. Transverse hippocampus sections (150 μm) were generated and mounted after staining. Images were first blinded, and the spines were manually counted and sorted.

### Primary neuron culture

Primary neurons were cultured as previously described [21] with slight modifications. Briefly, P0 newborn mice were euthanized before their hippocampi and cortices were dissected for further digestion. Papain (2 mg/ml) was used instead of trypsin. Cells for imaging analysis were plated on glass coverslips coated with poly-L-lysine (Sigma, P9155), while those destined for Western blot analysis were plated on poly-L-lysine coated plates. The cells were transfected with lentivirus at DIV1 using 10 MOI. The medium was changed 24 hours later. The medium was changed twice a week until the experiments were performed.

### Lentivirus packaging

HEK293T cells were used for lentivirus packaging. The pLKO.1-sh*Luc* (shRNA control plasmid), pHVSVG, and psPAX2 plasmids were donated from the Lim Lab [22]. The sequence design for sh*Prodh* was obtained using the webpage (www.sigmaaldrich.com/life-science/functional-genomics-and-rnai/sirna/mission-predesigned-sirna.html). The sequences of the primers for the sh*Prodh* constructs were 5’-CCG GTT ATG CCC AAG GCG GGA TTT CCT CGA GGA AAT CCC GCC TTG GGC ATA ATT TTT G-3’ and 5’-AAT TCA AAA ATT ATG CCC AAG GCG GGA TTT CCT CGA GGA AAT CCC GCC TTG GGC ATA A-3’. The DNA constructs (pLKO.1-sh*Luc* or pLKO.1-sh*Prodh*; pHVSVG, and psPAX2) were incubated in a 4:1:3 ratio and polyethylenimine for lentivirus production. The cell culture medium was changed ~16 hours after transfection and the medium containing the lentivirus particles was harvested 24 hours later. The harvested lentivirus particles were concentrated using Lenti-X^TM^ concentrator (Clonteck, PT4431-2) and the final virus titer was approximately 1×10^9^ TU/ml.

### Analysis of available databases

Schizophrenia-associated genes (Fig. 1A-B) and the expression of mRNAs involved in the proline metabolism pathway (Fig. 1E) [23] were obtained from SZDB2.0 (http://www.szdb.org/index.html) and DisGeNET (https://www.disgenet.org/search). Metabolic genes curated by the KEGG database were used for subsequent analysis. Genes with a Score_gda ≥ 0.1 in DisGeNET were included. Finally, 10 genes with at least 4 significant associations (total ≥ 4) in SZDB2.0 that were also found to be differentially expressed in the brains of schizophrenia patients were identified as metabolic genes related to schizophrenia. The association of these genes with schizophrenia was analyzed based on the following. (1) Brain expression: brain tissue gene expression data was obtained from The Human Protein Atlas. FPKM (fragments per kilobase million) was used as the measurement unit. (2) GWAS: whether a gene was identified by PGC2 GWAS or CLOZUK + PGC2 GWAS was recorded. (3) Linkage: whether a gene was identified by linkage and association studies was noted. (4) Integrative: genes identified by integrative analysis were recorded. (5) Diff Methy: genes that were differentially methylated in schizophrenia patients were obtained. (6) Exome: genes identified by exome sequencing were obtained. (7) GO: Gene Ontology annotations of genes related to brain or neuronal function were obtained. The gene-disease association analysis presented in Figure 1C was performed by DisGeNET. In Figure 1E, the logFC of mRNA expression denotes log_2_ (fold change), where fold change is the relative expression of a gene in schizophrenia cases compared to that observed in healthy controls. The differentially expressed genes in SZDB2.0 (Fig. 1F) were identified by CommonMind Consortium (CMC) by comparing the RNA sequencing data of 258 schizophrenia cases to 279 healthy controls. Post-mortem human brain specimens were obtained from three different brain banks: MSSM, Pitt, and Penn. More detailed patient information can be found in the 2016 CMC study [23]. The original data is available from www.synapse.org/CMC. The proline plasma concentrations of schizophrenia patients in Figure 1D were reanalyzed from a previously published study [24]. Gene expression levels in the prenatal and postnatal periods were analyzed by PsychENCODE Consortium (www.nimhgenetics.org/resources/psychencode#) [25].

### Statistical analysis

All data were analyzed using GraphPad Prizm and presented as the mean ± standard error of the mean (SEM). The normality of the data was determined by the Shapiro-Wilk test. If the data fit a normal distribution (*p* > 0.05), statistical differences were determined by two-way ANOVA or the two-tailed unpaired Student’s *t*-test, as indicated in the Figure legends. If the data were not normally distributed (*p* < 0.05 in the Shapiro-Wilk test), statistical differences were determined by Mann-Whitney test. Image J was used to conduct the neuron dendrite length measurements and intersection number analysis (Sholl analysis) [26].

## RESULTS

### Dysregulated proline metabolism in schizophrenia patients

Numerous genes were associated with schizophrenia. To identify potential metabolic pathways associated with schizophrenia, we performed comprehensive analyses of the gene expression data from two databases, SZDB2.0 and DisGeNET. Two hundred and thirty metabolic genes were identified to be schizophrenia-associated, of which only 10 were found to be common in both databases, including PRODH (Fig. 1A). In SZDB2.0, we found 10 metabolic genes, each of which had at least four significant associations (total ≥ 4) and altered expression in schizophrenia patients (Fig. 1B). Among the 10 on this list, PRODH and SACM1L had the highest expression levels in the human brain (Fig. 1B). On the other hand, schizophrenia was among the top 3 diseases associated with PRODH expression, including a specific subtype of schizophrenia known as schizophrenia 4 (Fig. 1C). These findings indicate that PRODH may play an important role in schizophrenia.

Consistently, the plasma proline concentration was higher in schizophrenia patients when compared to healthy controls [24] (Fig. 1D). To ascertain the role of proline metabolism in schizophrenia, we analyzed the expression of key proline degradation and biosynthesis genes in SZDB2.0 (Fig. 1E). The expression levels of two degradation enzymes, ALDH4A1 and PRODH, were lower in the brains of schizophrenia patients when compared to healthy controls (Fig. 1F). In contrast, the level of the proline synthesis enzyme PYCR1 was higher in schizophrenia patients, although two other proline synthesis enzymes, ALDH18A1 and PYCR2, exhibited slightly lower levels compared to healthy controls [23] (Fig. 1F). As schizophrenia commonly occurs at an early stage of life [27], we analyzed the expression levels of enzymes in the proline metabolic pathway during the pre-and post-natal period in humans. Consistently, the levels of proline degradation enzymes were greater and those of biosynthesis enzymes were lower at the postnatal stage compared to the prenatal stage (Fig. 1G), except for PYCR2 (Supplementary Fig. 1A). These data indicate that the proline level is reduced during the course of neurodevelopment. Taken together, the transcriptomic data indicate that the proline level should remain low in the mature nervous system, but is increased in the brains of schizophrenia patients.

### PRODH is broadly expressed in the brain, with enrichment in the dorsal hippocampus

**Figure 2.**
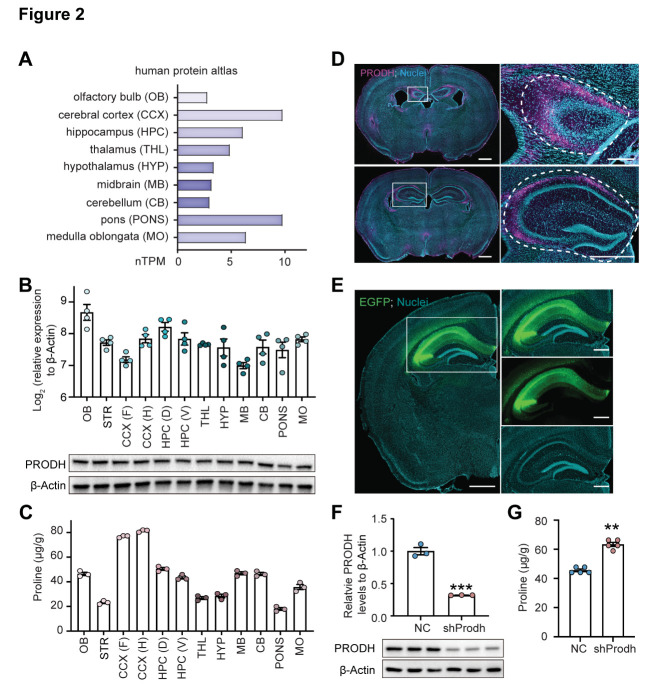
**Characterization of PRODH expression and proline levels across different brain regions**. (**A**) *Prodh* mRNA expression in 9 major human brain regions based on data obtained from the Human Protein Atlas. (**B**) *Prodh* mRNA and protein expression in 12 major mouse brain regions. N = 4. (**C**) Proline concentrations in the 12 major mouse brain regions. N = 3. (**D**) Representative images of PRODH immunofluorescence in mouse brain sections. Scale bar = 1 mm for the whole brain image (left panels), scale bar = 200 µm for the magnified view of the white box (right panels). (**E**) Representative image showing the stereotactic injection site. White box: mouse hippocampus. Left panel, scale bar = 1 mm; right panel, scale bar = 500 µm. F: The efficient KD of PRODH protein expression in the mouse hippocampus. N = 3 mice per group. The data are presented as the mean ± SEM. Statistical differences were determined by the two-tailed unpaired Student's *t*-test. ***p < 0.001. G: Elevated proline level in the hippocampus of PRODH KD mice. N = 5. The data were not normally distributed (*p <* 0.05 in the Shapiro-Wilk test). Statistical differences were determined by the Mann-Whitney test. **p < 0.01. CCX (F): frontal cerebral cortex; CCX (H): hind cerebral cortex; HPC (D): dorsal hippocampus; HPC (V): ventral hippocampus.

To investigate the role of PRODH and proline metabolism in brain function, we first analyzed the distributions and concentrations of PRODH and proline in human and mouse brains. For the human analysis, we examined the brain distribution of PRODH by analyzing the human protein atlas and found that PRODH was broadly expressed in the majority of brain regions, from the olfactory bulb to the medulla oblongata (Fig. 2A). For the study of mice brains, we conducted an expression analysis on mouse brain tissue using qPCR and Western blotting to find that PRODH was also broadly expressed in all the brain regions examined (Fig. 2B). We next measured the proline concentrations in the mouse brain, and indeed, proline was detected in all the examined brain regions (Fig. 2C). To identify the subregions with PRODH enrichment in the mouse brain, we performed immunofluorescent staining and found that the oreins layer of the hippocampus exhibited a relatively high expression of PRODH (Fig. 2D), although PRODH was also detected in the thalamus, midbrain, and other brain regions (Supplementary Fig. 1B). Meanwhile, the proline synthesis enzymes PYCR1 and PYCR2 were broadly distributed in the mouse brain without any specific regional enrichment (Supplementary Fig. 1C).

**Figure 3.**
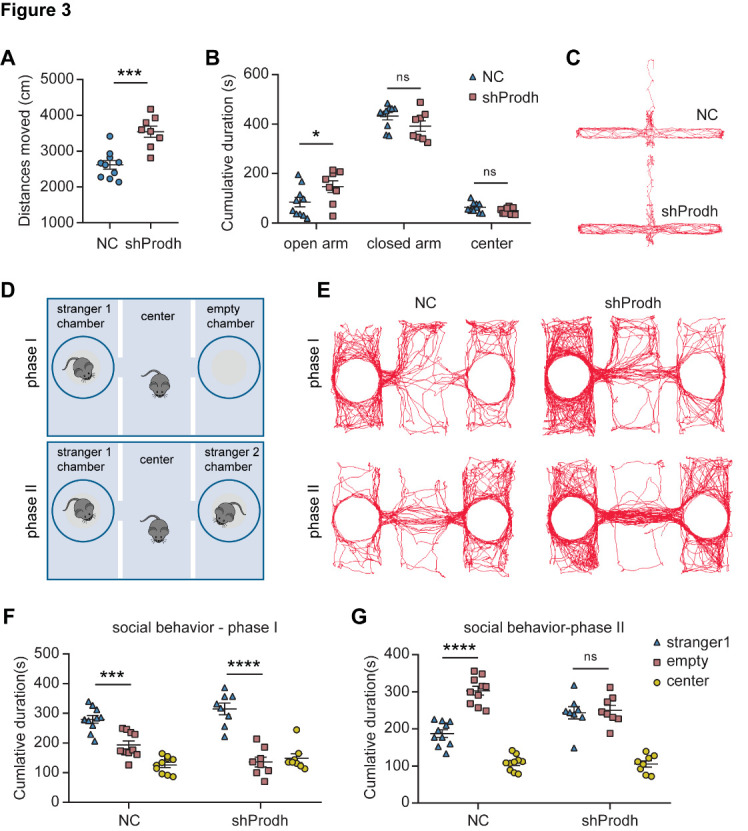
**Altered locomotor and social behaviors in mice with PRODH depleted in the hippocampus**. (**A-B**) Altered locomotor activities in PRODH KD mice, as measured by the distance moved (A); cumulative duration in each zone of the elevated maze (B). The data are represented as the mean ± SEM. Statistical differences were determined by the two-tailed unpaired Student's *t*-test. *p < 0.05, ***p < 0.001, ns: no statistical significance. (**C**) Representative images of mouse movement tracks in the elevated plus maze test. Closed arms were situated in the horizontal direction, while those of the open arms were vertical. (**D**) Three chamber social tests, including two phases, as shown in the diagram. (**E**) Representative images of mouse movement tracks in the three chambers during the social tests. (**F-G**) Cumulative duration spent in each chamber of phase I and phase II by the PRODH KD and control mice. The data are represented as the mean ± SEM. Statistical differences were determined by two-way ANOVA and the post hoc Tukey’s multiple comparison test. **p < 0.01, ***p < 0.001, ****p < 0.0001, ns: no statistical significance. All of the behavioral tests included 10 controls (NC) and 8 KD mice (shProdh).

Schizophrenia was reported to be closely associated with abnormalities in the structure of the hippocampus and the morphology of its neurons [3]. As PRODH was highly expressed in the hippocampus, we focused on this brain region. To do so, we first knocked down PRODH by administering a stereotaxic injection of AAV-sh*Prodh* to the dorsal hippocampus. We tested different virus injection volumes and found that a 700 nl volume containing the virus could cover a bigger area of the hippocampus compared to 300 nl, as expected (Supplementary Fig. 1D). We validated the infection and shRNA expression efficiencies by imaging EGFP fluorescence, which exhibited strong signals in the main region of the dorsal hippocampus (Fig. 2E). After conducting behavioral tests, the experimental mice were euthanized, and their brains were harvested for protein and metabolite quantification. PRODH expression was significantly reduced in the dorsal hippocampus, which is the target site of the AAV-sh*Prodh* injection (Fig. 2F, Supplementary Fig. 1E). Consistently, the proline concentration was significantly increased in these PRODH KD mice (Fig. 2G), which was also validated by HPLC-mass spectrometry (Supplementary Fig. 1F). These results show that PRODH was highly expressed in the dorsal hippocampus, which we were able to decrease alongside increasing the proline concentration in this brain region by performing the stereotaxic injection of AAV-sh*Prodh*.

### Altered locomotor and social behaviors in mice with reduced PRODH expression

To investigate the behavioral manifestations of PRODH depletion, we first examined the locomotor activity of PRODH KD mice using the open field test. The PRODH KD mice exhibited hyperactivity, as evidenced by significant increases in both their moving distance and speed over a 5-minute period when compared to the control mice (Fig. 3A, Supplementary Fig. 2A). Moreover, the PRODH KD mice did not show a preference for the border regions, but appeared to spend less time in the central zone (Supplementary Fig. 2B, S2C). We next examined anxiety-like behavior using the elevated plus maze test. The PRODH KD mice spent more time in the open arm compared to the control mice (Fig. 3B, 3C). No significant difference was observed in the visit frequency to the open arm between the PRODH KD and the control mice, although the PRODH KD mice had a much greater visit frequency to the closed arm and center (Supplementary Fig. 2D), as well as longer moving distances within the open and closed arms (Supplementary Fig. 2E), when compared to the control mice. Together, the open field test and the elevated plus maze test results demonstrate the hyperactivity phenotype of the PRODH KD mice.

Defects in social interaction and an increase in social withdrawal are core negative symptoms of schizophrenia that are particularly resistant to treatment [19]. We examined the social behavior of the PRODH KD mice by using the three-chamber sociability and social novelty tests [19]. In the first phase, both the PRODH KD and control mice spent more time in the side chamber containing a stranger mouse (stranger 1) compared to the chamber without one (Fig. 3D-F). The entry frequency of the PRODH KD mice to the stranger 1 chamber was significantly higher than that to the empty chamber, while the control mice did not show any difference in the entry frequencies to the two chambers (Supplementary Fig. 2F). In the second phase, a different stranger mouse (stranger 2) was placed in the empty cage. The control mice spent more time with stranger 2 and showed a higher entry frequency into the stranger 2 chamber when compared to that with stranger 1. In contrast, the PRODH KD mice did not exhibit any difference in the visitation duration or frequency between the two side chambers (Fig. 3E, G, Supplementary Fig. 2G). These results indicate that the PRODH KD mice had a reduced preference for social novelty.

### Impaired learning and memory in PRODH KD mice

**Figure 4.**
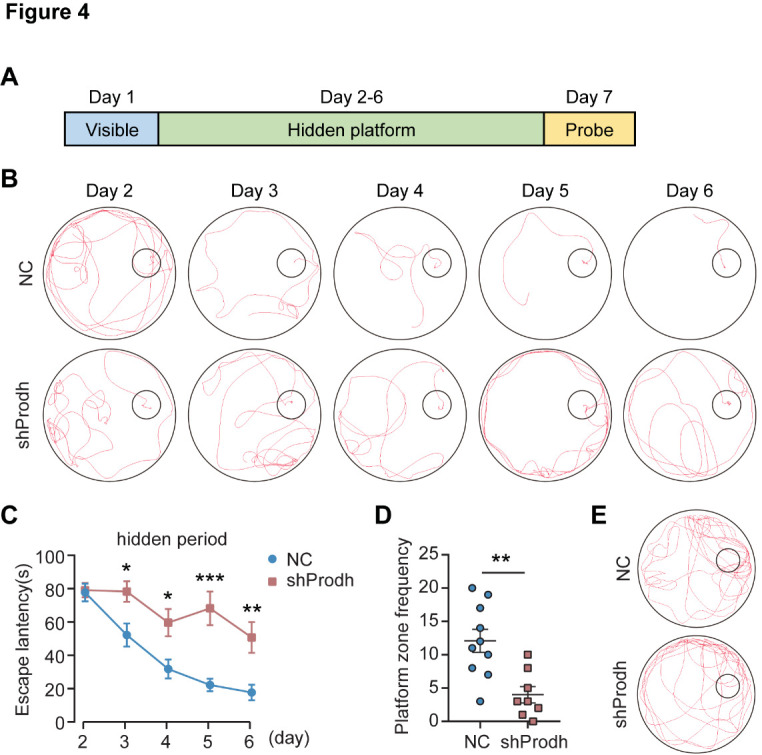
**Impaired learning and memory behaviors in mice with PRODH depleted in the hippocampus**. (**A**) Experimental design of the Morris water maze test. (**B**) Representative images of the Morris water maze test conducted over 5 consecutive days with a submerged escape platform. The smaller circle positioned inside marked the location of the hidden platform. (**C**) Comparison of the escape latency between the PRODH KD and control mice. The data are represented as the mean ± SEM. Statistical differences were determined by the two-tailed unpaired Student's *t*-test on individual days. *p < 0.05, **p < 0.01, ***p < 0.001. (**D**) Comparison of the frequency statistics of mouse entries to the platform zone. The data are represented as the mean ± SEM. Statistical differences were determined by the two-tailed unpaired Student's *t*-test on individual days. **p < 0.01. (**E**) Representative images of mouse movement tracks on the last probe day. The smaller circle inside marks the virtual platform location. All of the behavioral tests included 10 controls (NC) and 8 KD mice (shProdh).

To evaluate the effect of reduced PRODH expression in the hippocampus on long-term spatial learning and memory in mice, we performed the Morris water maze test [20] over 7 consecutive days. Day 1 involved platform-visible exclusion training to exclude the effects of vision and the fear of water in mice. Days 2-6 involved platform-hidden training to encourage the mice to learn how to locate the platform. Day 7 employed the probe test to assess the memory of mice (Fig. 4A). The maze was divided into 4 quadrants (Supplementary Fig. 3C), as previously described [20]. In the position navigation experiment, the mean escape latency of mice searching for a hidden platform decreased with the increase in training days (two-way ANOVA: days: *p* < 0.0001) (Fig. 3C). From days 3 to 6, the PRODH KD mice exhibited a significantly longer escape latency compared to the control mice (Fig. 4C). The swimming path was also significantly longer for the PRODH KD mice than the control mice (Fig. 4B, Supplementary Fig. 3A). Consistently, the escape speed of the PRODH KD mice was significantly lower than the control mice on days 5 and 6 (Supplementary Fig. 3B). In the probe phase, the hidden platform was removed, and a probe test was performed on day 7 to assess the memory recall of the mice. The probe test showed that mice injected with AAV-sh*Prodh* spent a similar amount of time as the control mice in the target quadrant and other three quadrants (Supplementary Fig. 3D). However, the frequency of entries recorded in mice injected with AAV-sh*Prodh* was significantly lower than that of control mice in the platform zone (Fig. 4D-E). These results suggest that the downregulation of PRODH expression in the mouse hippocampus impairs spatial learning, lowers spatial reference, and impacts certain aspects of cognitive plasticity in mice.

**Figure 5.**
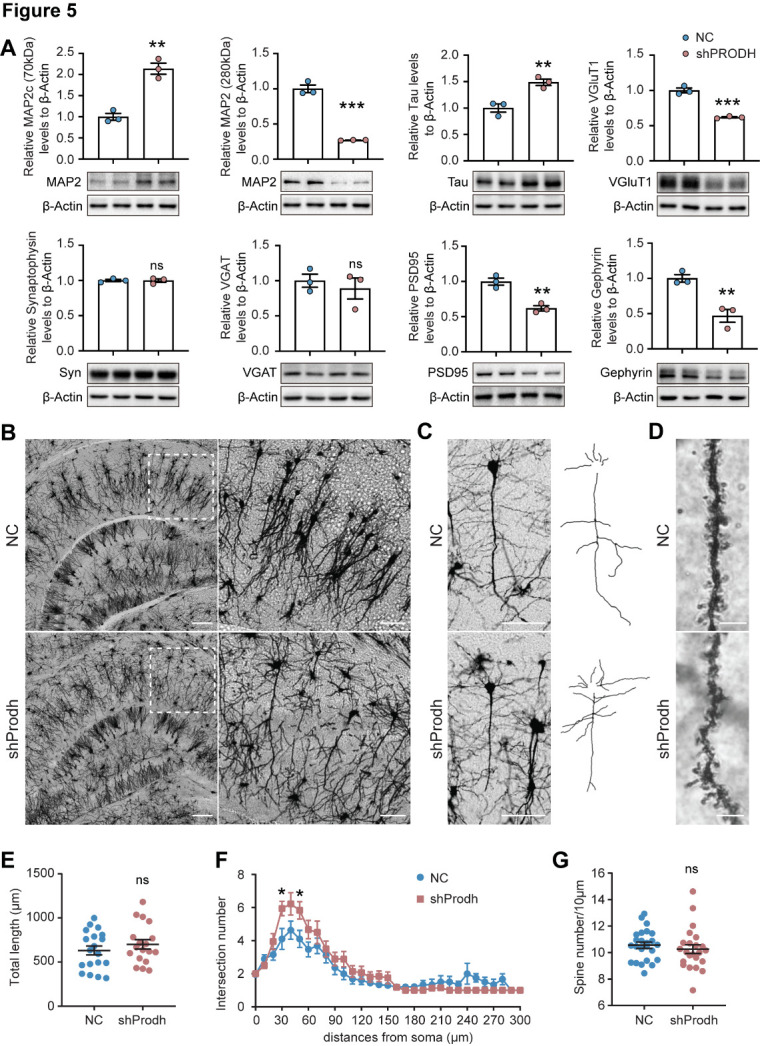
**The KD of PRODH alters neuronal morphology in the hippocampus**. (**A**) The protein expression levels of MAP2, Tau, VGluT1, Synaptophysin (Syn), VGAT, PSD95, and Gephyrin between the NC and shProdh mouse hippocampus. N = 3. The data are represented as the mean ± SEM. Statistical differences were determined by the two-tailed unpaired Student's *t*-test. **p < 0.01, ***p < 0.001, ns: no statistical significance. (**B**) Representative images showing the Golgi-cox staining results of NC and shProdh mice. The scale bar of the first row of images is 200 µm; the scale bar of the second row of images is 100 µm. The second-row images show the magnified area of the white square box images displayed in the first row. (**C**) Representative images showing the Golgi-cox staining results for a single pyramidal cell from each of the NC and shProdh mice. Scale bar: 100 µm. The right images are the cell paths’ snap pictures. (**D**) Representative images showing the Golgi-cox staining results of the spine density of NC and shProdh mice. Scale bar: 5 µm. E-G: Statistics of the pyramidal cells’ length, intersection numbers (analyzed by Sholl analysis), and spine density in NC and shProdh mouse hippocampus CA regions. N(NC) = 18, N(shProdh) = 19. The data are represented as the mean ± SEM. Statistical differences were determined by the two-tailed unpaired Student's *t*-test. *p < 0.05, ns: no statistical significance.

**Figure 6.**
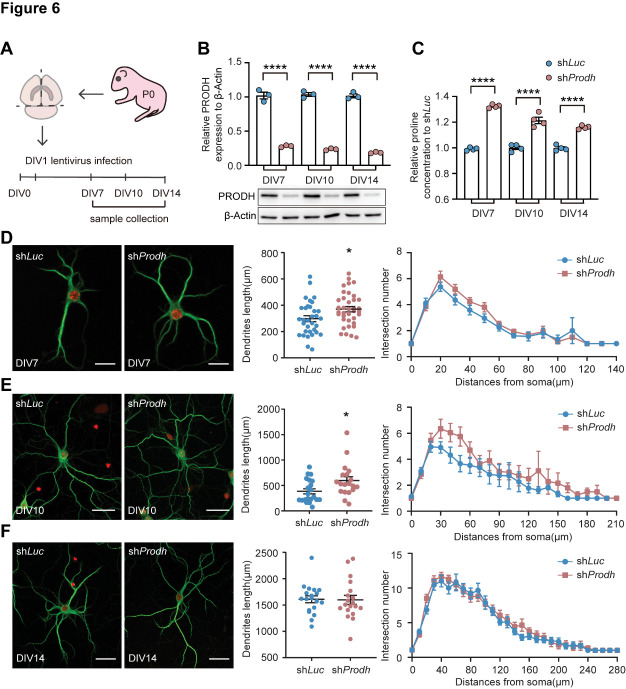
**The KD of PRODH changed the morphology and function of neurons *in vitro.* (A) Schematic diagram of P0 newborn mouse primary neuron culture**. (**B**) Statistics of the PRODH expression level at DIV7, DIV10, and DIV14. N = 3. The data are represented as the mean ± SEM. Statistical differences were determined by the two-tailed unpaired Student's *t*-test on each day. ****p < 0.0001. (**C**) Statistics of the proline concentration in PRODH KD cultured cells compared to controls at DIV7, DIV10, and DIV14. N = 4. The data are represented as the mean ± SEM. Statistical differences were determined by the two-tailed unpaired Student's *t*-test on each day. ****p < 0.0001. (**D-F**) Representative staining of MAP2 to mark the dendrites of neurons at DIV7, DIV10, and DIV14. Scale bar (DIV7): 20 µm; scale bar (DIV10, 14): 40 µm. Statistics of the total dendrite length and intersection numbers. DIV7: N(sh*Luc*) = 33, N(sh*Prodh*) = 38; DIV10: N(sh*Luc*) = 22, N(sh*Prodh*) = 20; DIV14: N(sh*Luc*) = 19, N(sh*Prodh*) = 19. The data are represented as the mean ± SEM. Statistical differences were determined by the two-tailed unpaired Student's *t*-test (D). The data were not normally distributed (*p <* 0.05 in the Shapiro-Wilk test). Statistical differences were determined by the Mann-Whitney test (E). *p < 0.05.

### Altered morphology and function of neurons in the PRODH KD hippocampus

To further elucidate the molecular mechanisms underpinning the observed cognitive dysfunction in the PRODH-deficient mice, we examined the expression of neuron-related markers in the dorsal hippocampus. Surprisingly, the neuron dendrite marker MAP2 showed an opposite trend of expression change for the 280 kDa and 70 kDa isoforms (Fig. 5A). The MAP2c (70 kDa) isoform has been reported to play an important role in extended neurites, similar to that attributed to the neuron axon marker that is mature Tau, while the MAP2 (280 kDa) isoform is known to mainly function in mature dendrites [28]. In the dorsal hippocampus where PRODH was knocked down, the levels of MAP2c and Tau were significantly increased, while the mature dendrite marker MAP2 was significantly decreased (Fig. 5A) compared to the control mice. These results indicate the overgrowth of both dendrites and axons in PRODH KD neurons. To further characterize the morphology of the neurons, we examined the expression of synaptic markers. The expression levels of the excitatory pre- and post-synaptic markers VGluT1 and PSD95 were significantly decreased in the dorsal hippocampus of the PRODH KD mice. The expression of the inhibitory post-synaptic marker Gephyrin was significantly decreased in the PRODH KD mice, while the expression levels of synaptophysin and the inhibitory presynaptic marker VGAT were unaltered (Fig. 5A). We next examined the expression of these proteins in the frontal cerebral cortex (PFC), which is another important brain region implicated in schizophrenia [29]. The expression of PRODH was slightly reduced, but not significantly, in the PRODH KD mice compared to the controls (Supplementary Fig. 4). The MAP2 and VGAT levels increased significantly in the PFC, suggesting that hippocampal PRODH deficiency also impacts the expression of neuron markers in other brain regions (Supplementary Fig. 4).

We next examined the morphology of the neurons by performing Golgi staining on the hippocampus, finding that the arrangement of neuronal dendrites was disordered in the hippocampal CA1 region, but not in the CA3 region (Fig. 5B). We then analyzed the neurite length and spine density of pyramidal cells in mouse hippocampal CA regions (Fig. 5C-G). Mice injected with AAV-sh*Prodh* did not show any significant difference in neurite length compared to the control mice (Fig. 5C, E). However, Sholl analysis [26] revealed an increased number of intersections between 30 and 50 µm away from the soma in mice injected with AAV-sh*Prodh* (Fig. 5C, F). We also did not observe any differences in the spine density between the control and PRODH KD mice (Fig. 5D, G). The Golgi staining results showed a slight increase in the intersection numbers of pyramidal neurons in mouse hippocampal CA regions. Considering the protein expression findings, we hypothesize that the KD of PRODH in the mouse hippocampus stimulates new protrusion formation and neurite overgrowth, consequently disrupting pyramidal neuron dendrite organization and function.

### Altered morphology and function of PROHD-depleted neurons

To ascertain changes in the morphology of neurons, we tracked the neurite formation process using primary neuron culture and used lentivirus-mediated shRNA to knock down PRODH. After neurons were isolated and plated in a dish, they were infected with lentivirus at DIV1 and incubated for 24 h. Neuron samples were collected at DIV7, DIV10, and DIV14 (Fig. 6A). PRODH was significantly reduced at each time point and the proline concentration increased accordingly (Fig. 6B, C). Furthermore, we used MAP2 as a dendrite marker to examine the morphological changes in neurons. At DIV7 and DIV10, the dendrite lengths were significantly increased, although the number of intersections was unaltered (Fig. 6D, E). Finally, no difference in the dendrite length or intersection number was observable at DIV14, when the neurons were mature (Fig. 6F). These findings indicate that the KD of PRODH affects the development of neuron dendrites, which is supported by the earlier observation of neuronal overgrowth and disarrangement *in vivo*.

We also examined the protein expression of MAP2, Tau, VGluT1, PSD95, VGAT, and Gephyrin in DIV10 cultured neurons. As was consistent with the *in vivo* experimental data, Tau expression was significantly increased, while the expression levels of VGluT1, VGAT, Gephyrin, and PSD95 were decreased. Interestingly, GAP43, an axon guidance protein, was significantly increased (Supplementary Fig. 5). Taken together, PRODH expression appears to be essential in the maintenance of neuronal morphology and function both *in vivo* and *in vitro*.

Neurotransmitters play a crucial role in brain function, and numerous studies have indicated that alterations in brain neurochemicals can impact neurotransmitter levels to subsequently cause behavioral changes. The levels of neurotransmitters were measured in the DIV10 cultured neurons. However, while 16 neurotransmitters were detected; including GABA, glutamine, glutamate, and so on; none showed a significant difference between the PRODH knockdown cells and shLuc controls (Supplementary Fig. 6). These results indicate that PRODH knockdown did not significantly change neurotransmitter levels *in vitro*.

## DISCUSSION

### Identification of proline degradation brain regions associated with psychiatric disorders

Current treatments for schizophrenia are largely limited to providing symptom control without addressing the underlying pathology [30]. It has long been recognized that structural aberrations of the hippocampus are a common hallmark of schizophrenia [3]. Hippocampal abnormalities in schizophrenia patients include reduced hippocampal volume, dysregulated levels of synaptic proteins, and impaired connectivity between the hippocampus and other parts of the central nervous system [3]. The hippocampus is associated with spatial learning and memory [31]. Based on anatomical connectivity and behavioral output, the rodent hippocampus can be divided into its dorsal and ventral parts [32]. Analogous to the human posterior hippocampus, the rodent dorsal hippocampus receives exteroceptive information from the entorhinal cortex and plays an important role in rapid spatial learning [33]. In contrast, the ventral hippocampus, which is analogous to the human anterior hippocampus, receives interoceptive information through interconnections formed with limbic regions that modulate motivational and affective states [34]. In our study, we first mapped the expression of the schizophrenia risk gene encoding PRODH and identified its specific enrichment in the oriens layer of the dorsal hippocampus. This finding not only indicates that PRODH is involved in rapid spatial learning, as evidenced by the Morris water maze test, but also suggests that the oriens layer of the dorsal hippocampus, which contains PRODH+ neurons, is potentially a subregion involved in the development of schizophrenia. Furthermore, the oriens layer of the hippocampus consists of interneurons [35], which gives new insight into the connection between PRODH and interneurons and can guide future studies. In addition to the hippocampus, other brain regions with high PRODH expression; such as the frontal cortex, striatum, and thalamus; are candidates for further studies [36, 37].

### Proline metabolism plays an important role in schizophrenia

A previous study that performed functional enrichment analysis using a microarray of drug-naive and drug-treated schizophrenia participants revealed that proline metabolism was dysregulated and that both arginine and proline metabolism were the most functionally enriched pathways for schizophrenia [38]. Jacquet *et al*. reported that three patients with schizophrenia had hyperprolinemia arising from the accumulation of proline in the blood [14]. Huggard *et al*. reported a case of a patient with hyperprolinemia type II with developmental delay and schizophrenia [39]. These patients did not have mutations in PRODH, indicating that proline accumulation can independently lead to schizophrenia without the presence of genetic defects. Furthermore, Crabtree and Gogos proposed that L-proline structurally mimics the neurotransmitters gamma-aminobutyric acid (GABA) and glutamate, and furthermore, that hyperprolinemia may cause interference with GABA-binding sites [40]. Herein, we found that the accumulation of proline *in vivo* and *in vitro* affected the morphology and function of neurons, which then induced schizophrenia-like behaviors in mice. Taken together, proline accumulation, whether or not it is the result of PRODH deficiency, may induce schizophrenia-like behaviors.

### PRODH is associated with schizophrenia-like behaviors

PRODH is the first degradation enzyme in the proline metabolism pathway. It has been identified as a schizophrenia susceptibility gene located at chromosome 22q11.2 [16]. PRODH knockout mice, which were originally crossed between the 129/ReJ and C57BL/6J strains, have been reported to exhibit significant attenuation in the overall level of prepulse inhibition [41], which is a reliable index for probing the neurobiology and genetics of gating deficits in schizophrenia both in humans and rodents [42]. PRODH knockout mice have also been reported to exhibit decreased locomotor activity compared to their wild-type littermates [43]. In our study, the KD of PRODH in the mouse dorsal hippocampus led to hyper-locomotion, suggesting a hippocampus-specific role of PRODH. The hyper-locomotion phenotype is more consistent with schizophrenia-like behaviors compared to decreased locomotor activity. To our knowledge, this is the first report to offer characterizations of the social behavior, learning ability, and memory ability of PRODH-deficient mice. Mice with PRODH KD in the hippocampus showed less interest towards a stranger mouse compared to controls, as well as worse learning and memory behaviors. All of the observed behavioral defects reported herein are similar to those observed in the schizophrenia mouse model established using glutamatergic manipulation [39], further indicating the PRODH KD mouse exhibits schizophrenia-like behavior. It should be noted that the gender and age of the mice used in the current study impose certain limitations and that the model does not perfectly imitate the onset period of human schizophrenia patients. We also showed in this study that the expression levels of VGluT1 and PSD95 were reduced. Taken together, these results can be collated to argue that PRODH affects the behavior of mice through modulating glutamatergic neurons, but the underlying mechanism requires further study.

### PRODH induces schizophrenia-like behaviors by altering neuronal morphology and function

Previous studies have not examined whether PRODH deficiency affects neuronal morphology. Herein, we show that PRODH plays an important role in modulating neuronal morphology both *in vivo* and *in vitro*. The expression levels of both excitatory and inhibitory neuron biomarkers were decreased when PRODH was knocked down, which is consistent with observations in schizophrenia patients [44]. *In vitro*, the axon guidance protein GAP43 was significantly increased during neurite growth. However, GAP43 expression was unchanged when the primary neuron matured at DIV14 in cell culture. Interestingly, existing data on the expression level of GAP43 in schizophrenia patients has been controversial [45-48]. The finding that GAP43 expression changes at DIV10 indicates that PRODH may influence neuron neurite growth during development. Overall, our results suggest that PRODH induces schizophrenia-like behaviors by altering neuronal morphology and function. On the other hand, PRODH knockdown led to proline accumulation. It is hard to prove that proline metabolism is the main pathway responsible for altering neuronal function and causing abnormal behavior. Further metabolic evidence is needed in future studies.

In conclusion, we report that PRODH deficiency leads to changes in neuronal morphology and function, which then leads to behavioral abnormalities in mice. These findings highlight the importance of proline metabolism in the development of schizophrenia. This area of study is expected to attract significant future attention.

## Supplementary Materials

The Supplementary data can be found online at: www.aginganddisease.org/EN/10.14336/AD.2023.0902.


