# Supplementary material for: Schizophrenia-Like Behaviors Arising from Dysregulated Proline Metabolism Are Associated with Altered Neuronal Morphology and Function in Mice with Hippocampal PRODH Deficiency
Source: Aging Dis. 2024 Aug 1;15(4):1952–68. doi: 10.14336/AD.2023.0902 (PMC11272211; doi:10.14336/AD.2023.0902)
Supplement: Supplementary file 1 [file AD-15-4-1952-s.pdf]

SUPPLEMENTARY DATA

Schizophrenia-Like Behaviors Arising from Dysregulated Proline Metabolism Are Associated with Altered Neuronal Morphology and Function in Mice with Hippocampal PRODH Deficiency

Yuxiao Yao, Chenchen Jin, Yilie Liao, Xiang Huang, Ziying Wei, Yahong Zhang, Dongwei Li, Huanxing Su, Weiping Han, Dajiang Qin

© 2023. Yao Y et al. Published online at http://www.aginganddisease.org/EN/10.14336/AD.2023.0902

SUPPLEMENTARY DATA

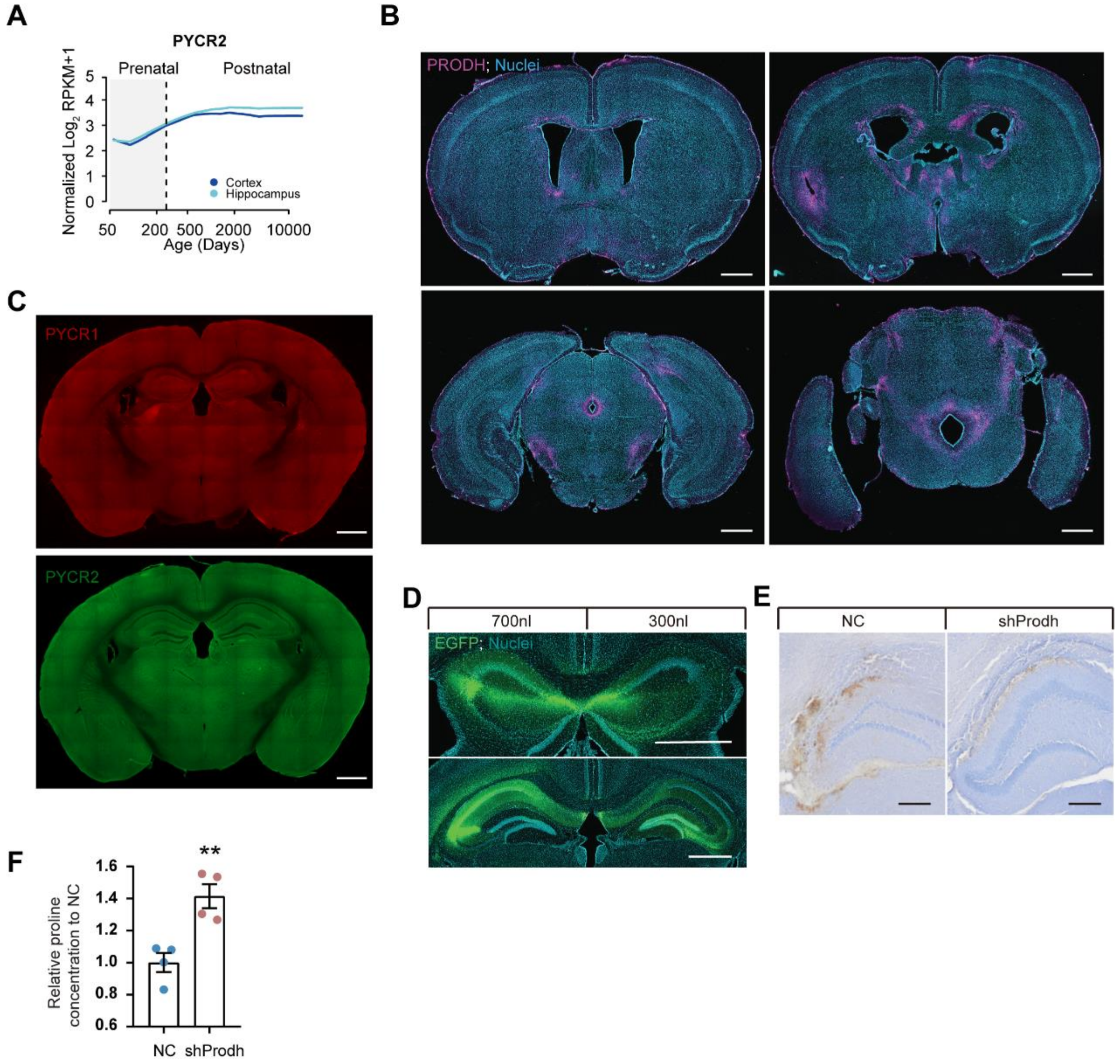

Supplementary Figure 1. PRODH expression pattern in mouse brain. A: PYCR2 expression in human cortex and hippocampus over the entire span of neurodevelopment. **B:** Representative images of PRODH immunofluorescence in mouse brain coronal sections. Scale bar: 1 mm. **C:** Representative images of PYCR1 and PYCR2 immunofluorescence in mouse brain coronal sections. Scale bar: 1 mm. **D:** Representative images of the left and right mouse brain hippocampus after stereotactic injection with 700 nl or 300 nl AAV-shProdh respectively. Scale bar: 1 mm. **E:** Immunohistochemistry staining with paraffin fixation in normal control (NC) and shProdh samples. Scale bar: 500 µm. **F:** Relative proline concentration in the shProdh goup as compared to NC. N = 4. Data are represented as mean ± SEM, and statistical differences are determined by two-tailed unpaired Student's *t*-test.

SUPPLEMENTARY DATA

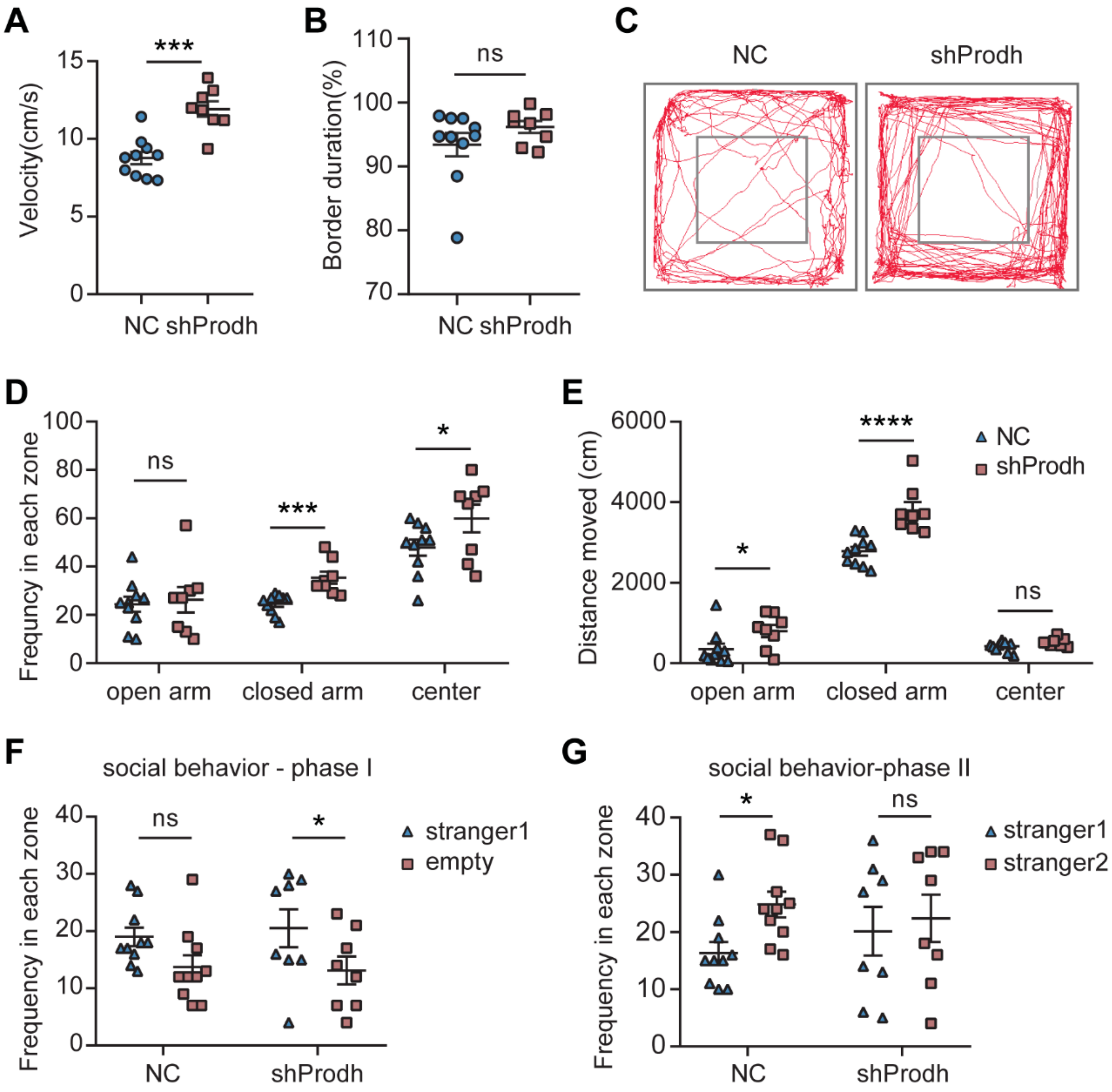

Supplementary Figure 2. Altered locomotor and social behaviors in PRODH-deficient mice. A-B: Measurements of local motor activity in movement velocity (A) and border duration (B) in PRODH KD (shProdh) and control (NC) mice. Data are represented as mean ± SEM, and statistical differences are determined by two-tailed unpaired Student's *t*-test. ***$p<0.001$, ns: no significant difference. **C:** Representative images of mouse movements in locomotion experiments. **D-E:** Frequency (D) and distance (E) moved in each zone of the elevated plus maze. Data are represented as mean ± SEM, and statistical differences are determined by two-tailed unpaired Student's *t*-test. *$p<0.05$, ***$p<0.001$, ****$p<0.0001$, ns: no significant difference. **F-G:** Frequency of movements in each chamber of phase I (F) and phase II (G) for shProdh and NC mice. Data are represented as mean ± SEM, and statistical differences are determined by two-tailed unpaired Student's *t*-test. *$p<0.05$, ns: no significant difference. All of the behavior tests include 10 NC and 8 shProdh mice.

© 2023. Yao Y et al. Published online at http://www.aginganddisease.org/EN/10.14336/AD.2023.0902

SUPPLEMENTARY DATA

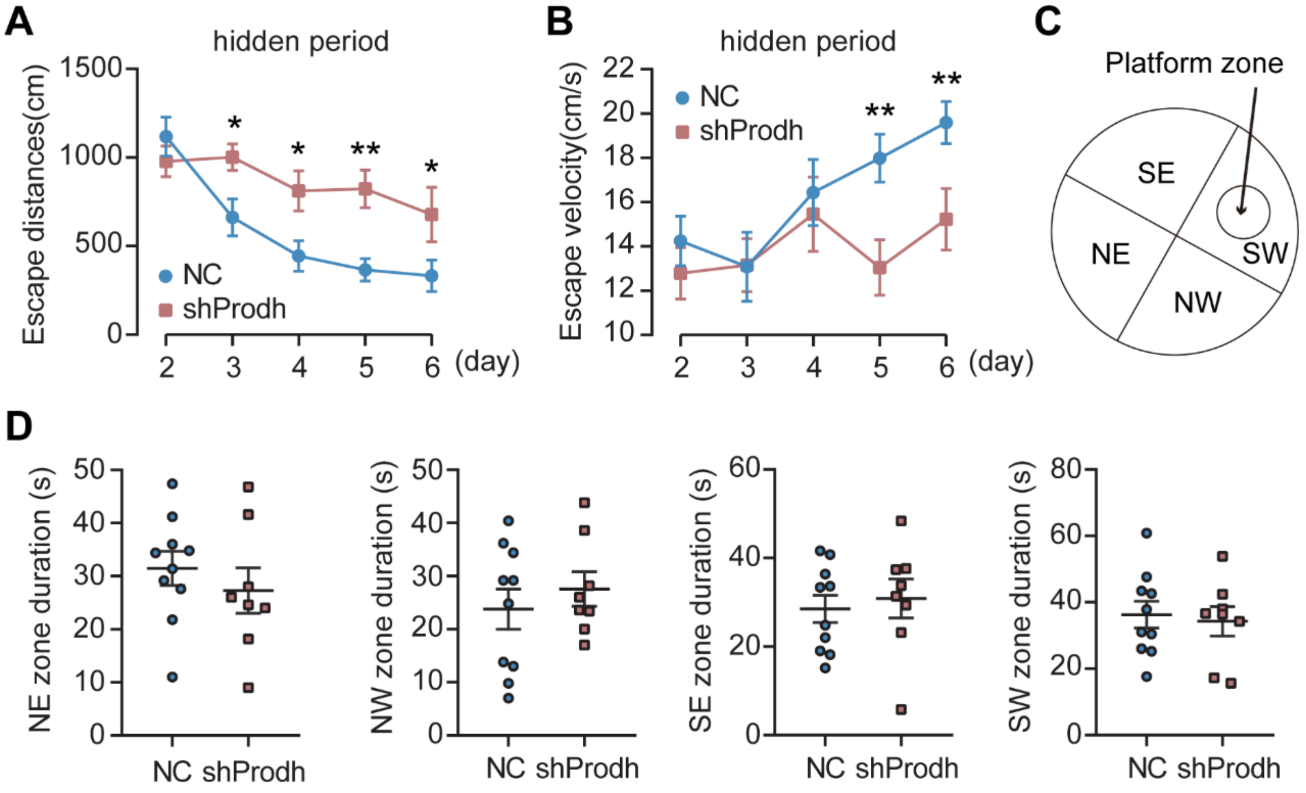

Supplementary Figure 3. Altered learning and memory behaviors in PRODH KD mice. A-B: Escape distance (A) and velocity (B) during the hidden platform period for PRODH KD (shProdh) and control (NC) mice. Data are represented as mean ± SEM, and statistical differences are determined by two-tailed unpaired Student's *t*-test for each day. **p*<0.05, ***p*<0.01. **C:** Schematic diagram showing 4 equal quadrants of the Morris water maze. The platform zone was in the SW quadrant. **D:** Duration of stay in each quadrant for shProdh and NC mice. There was no significant difference in any of the four quadrants. All of the behavior tests include 10 controls (NC) and 8 PRODH KD mice (shProdh).

© 2023. Yao Y et al. Published online at http://www.aginganddisease.org/EN/10.14336/AD.2023.0902

SUPPLEMENTARY DATA

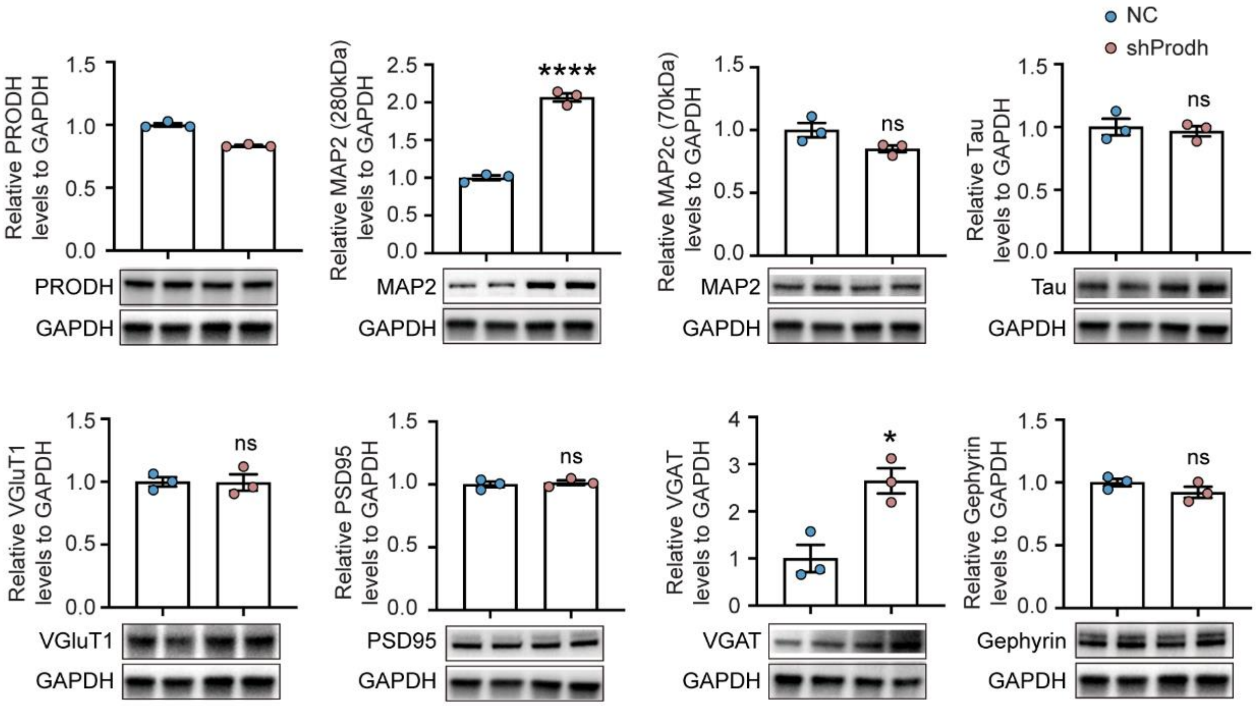

Supplementary Figure 4. Altered expression of targeted proteins in the prefrontal cortex of PRODH-deficient mice. Expression levels of targeted proteins, including PRODH, MAP2, Tau, VGluT1, PSD95, VGAT, and Gephyrin, were examined in the prefrontal cortex of control (NC) and PRODH-KD (shProdh) mice. Data are represented as mean ± SEM, and statistical differences are determined by two-tailed unpaired Student's *t*-test. Since PRODH data are not normally distributed (p <0.05 in Shapiro-Wilk test), the statistical difference is determined by Mann-Whitney test. ns: no significant difference, *p<0.05, ****p<0.0001. N = 3 per group.

© 2023. Yao Y et al. Published online at http://www.aginganddisease.org/EN/10.14336/AD.2023.0902

SUPPLEMENTARY DATA

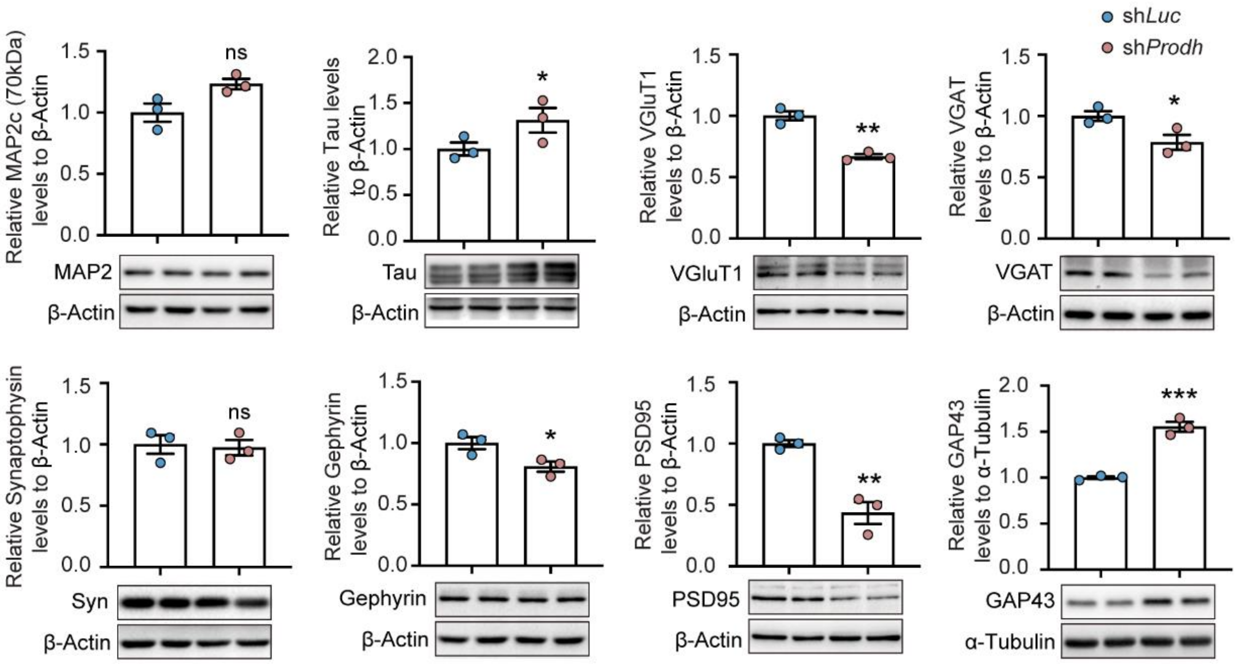

Supplementary Figure 5. Altered expression of targeted proteins in PRODH KD neurons *in vitro.* Expression levels of selected proteins, including MAP2, Tau, VGluT1, PSD95, VGAT, Gephyrin, Synaptophysin, and GAP43, were examined in PRODH KD cultured cells (sh*Prodh*) and controls (sh*Luc*). Data are represented as mean ± SEM, and statistical differences are determined by two-tailed unpaired Student's *t*-test. ns: no significant difference, *p<0.05, **p<0.01, ***p<0.001. N = 3 per group.

© 2023. Yao Y et al. Published online at http://www.aginganddisease.org/EN/10.14336/AD.2023.0902

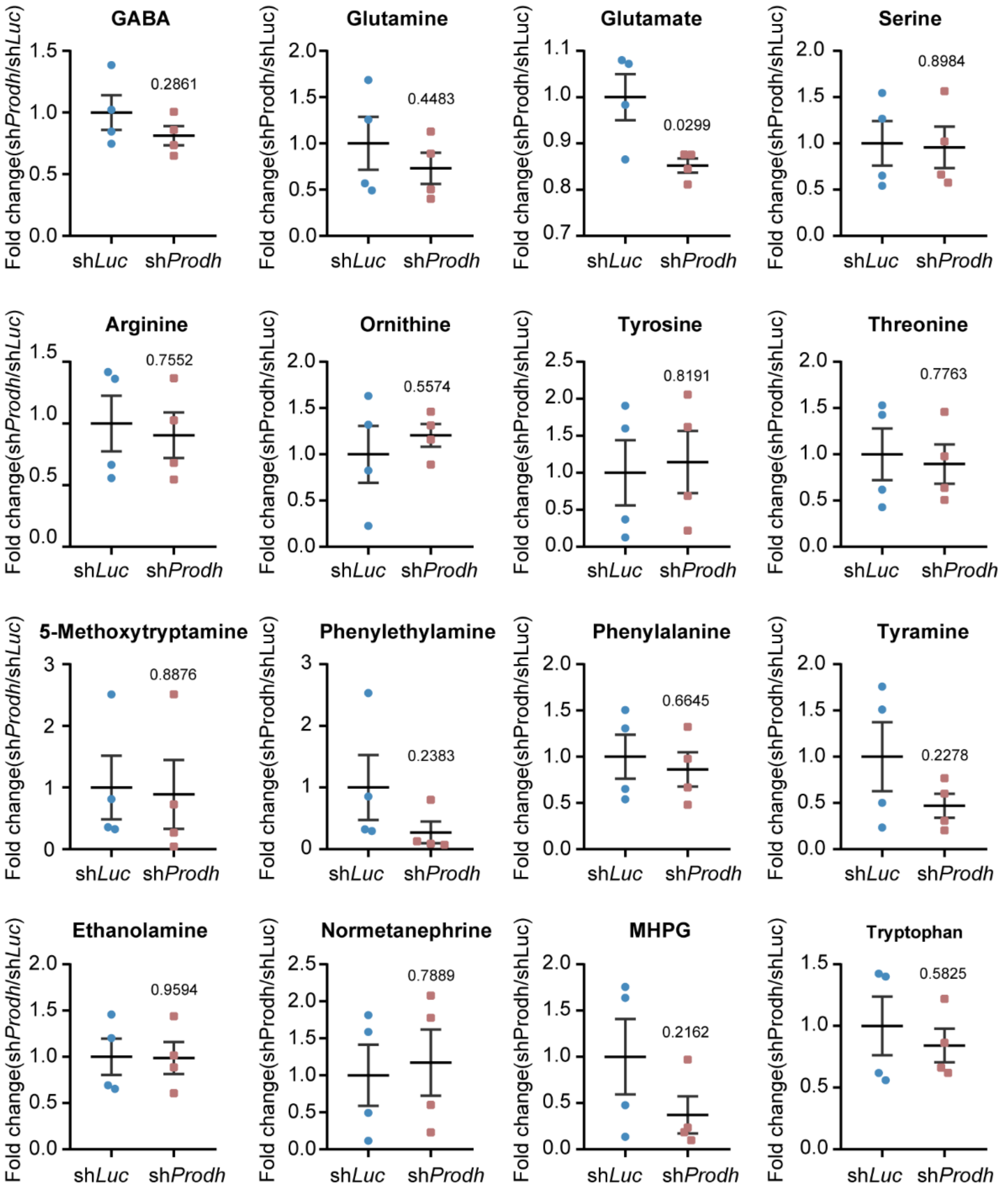

Supplementary Figure 6. Neurotransmitter in PRODH KD neurons *in vitro*. Neurotransmitters were examined in PRODH KD neurons (sh*Prodh*) and controls (sh*Luc*). Data are represented as mean ± SEM, and statistical differences are determined by two-tailed unpaired Student's *t*-test. N = 4 per group. All showed no significant differences.

© 2023. Yao Y et al. Published online at http://www.aginganddisease.org/EN/10.14336/AD.2023.0902

SUPPLEMENTARY DATA

Supplementary Table 1. Detailed information on the antibodies in the study.

Antibody name	Company	Catalog
mouse anti-PRODH	Santa Cruz	sc-376401
mouse anti-MAP2	Santa Cruz	sc-74421
mouse anti-Tau	Cell Signaling Technology	4019
mouse anti-VGluT1	Abcam	ab242204
Rabbit anti-Synaptophysin	Cell Signaling Technology	36406
Rabbit anti-VGAT	Thermo Fisher	PA5-27569
Rabbit anti-PSD95	Proteintech	20665-1-AP
Rabbit anti-Gephyrin	Thermo Fisher	PA5-29036
Rabbit anti-GAP43	Cell Signaling Technology	8945

© 2023. Yao Y et al. Published online at http://www.aginganddisease.org/EN/10.14336/AD.2023.0902
